# Synthesis of a bulk nanostructured metastable Al alloy with extreme supersaturation of Mg

**DOI:** 10.1038/s41598-019-53614-3

**Published:** 2019-11-20

**Authors:** Jae-Kyung Han, Klaus-Dieter Liss, Terence G. Langdon, Megumi Kawasaki

**Affiliations:** 1 School of Mechanical, Industrial and Manufacturing Engineering, Oregon State University, Corvallis, OR 97331 USA; 2 Materials and Engineering Science Program, Guangdong Technion - Israel Institute of Technology, Shantou, Guangdong 515063 China; 3 Technion – Israel Institute of Technology, Haifa, 32000 Israel; 4 Materials Research Group, Department of Mechanical Engineering, University of Southampton, Southampton, SO17 1BJ UK

**Keywords:** Metals and alloys, Design, synthesis and processing

## Abstract

Nanostructuring of bulk metals is now well documented with the development of severe plastic deformation (SPD) for improving the physical and mechanical properties of engineering materials. Processing by high-pressure torsion (HPT), which was developed initially as a grain refinement technique, was extended recently to the mechanical bonding of dissimilar metals during nanostrcturing which generally involves significant microstructural heterogeneity. Here we introduce, for the first time, a bulk metastable Al-Mg supersaturated solid solution by the diffusion bonding of separate Al and Mg metal solids at room temperature using HPT. Exceptional hardness was achieved homogeneously throughout the metastable alloy with a record maximum supersaturated Mg content of ~38.5 at.% in the Al matrix having a grain size of ~35–40 nm. Our results demonstrate the synthesis of a bulk nanocrystalline metastable alloy with good microstructural stability at room temperature where such bulk solids are not yet reported for mechanical alloying by powder metallurgy.

## Introduction

An understanding of the principles and the technical parameters of many different SPD techniques has provided an opportunity to introduce superior mechanical properties and additional functionalities in nanostructured materials^1–4^. Among the available SPD techniques, HPT is recognized as one of the most attractive methods since significant grain refinement is anticipated by comparison with other SPD procedures and often the technique is effective for hard-to-deform materials even at room temperature^5^. Utilizing this benefit, a new approach to mechanical bonding by HPT was examined recently for the synthesis of bulk hybrid nanostructured alloys by layering dissimilar nanostructured metals^6–8^ and ultimately leading to metal matrix nanocomposites by forming a reinforcement of a few nano-scale intermetallic phases^9–14^. In practice, this procedure applies the conventional HPT technique concurrently on two or more different kinds of bulk metals. The microstructures in the mechanically bonded metal systems by HPT processing generally develop into heterostructures^15^ involving heterogeneous and gradient microstructures from the sample surfaces to the depth. Thus, no clear information is available currently on any microstructural saturation and an ultimate metal state, thereby effectively limiting the mechanical properties when mechanically bonding dissimilar metals by HPT after very severe deformation. In this study, Al and Mg disks were mechanically bonded at room temperature by HPT for shear strains up to 4,000 through 100 torsional turns to assess the asymptotic microstructure by applying the simple yet powerful method of X-Ray diffraction (XRD) peak profile analysis.

**Figure 1:**
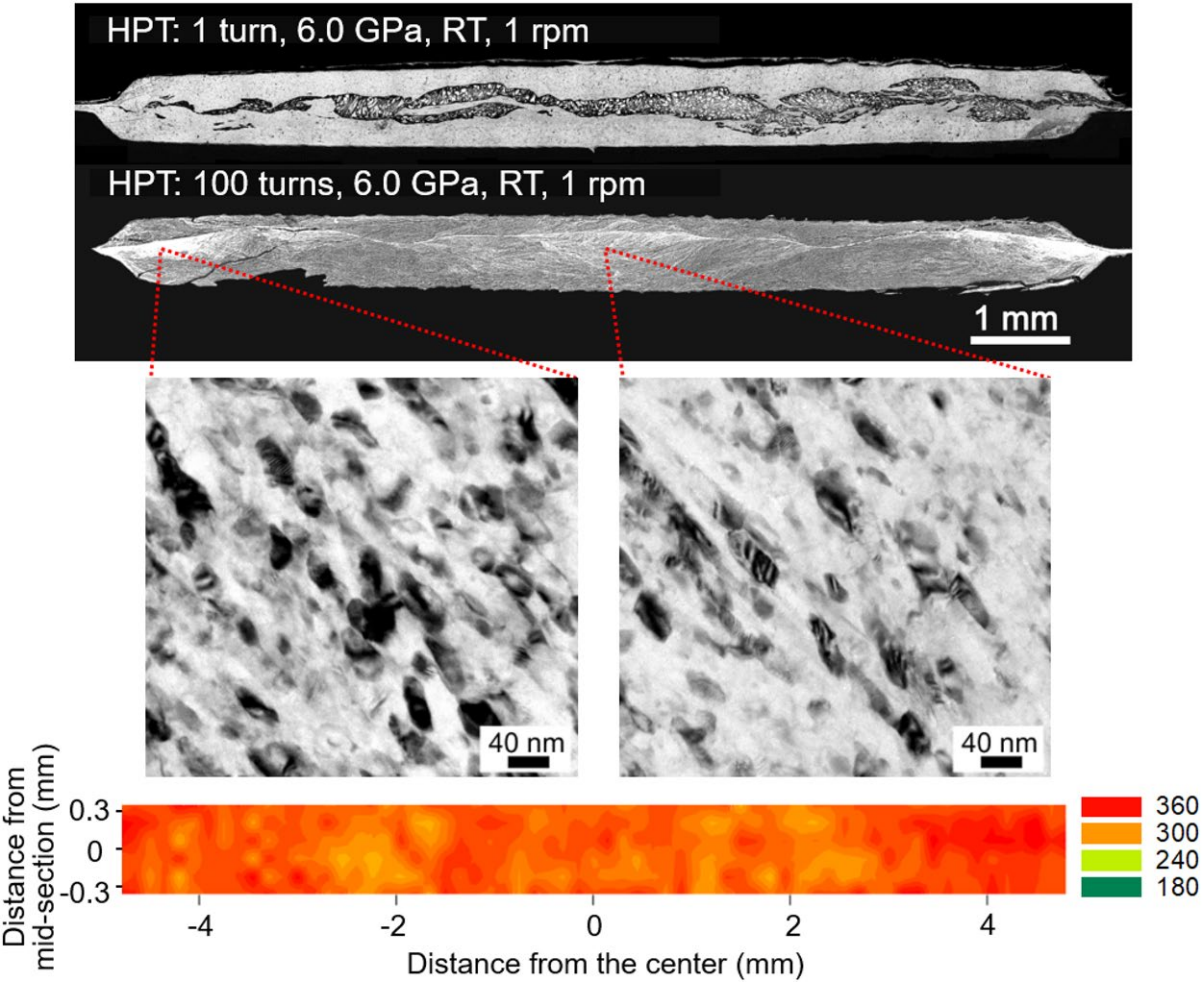
Deformed microstructure and the exceptional hardness of an Al-Mg system after HPT. The upper micrographs show the cross-sections of the Al-Mg disks after HPT for 1 turn as an example of mechanical bonding^9^ and 100 turns. In the center, the TEM micrographs taken at the disk center and edge prove microstructural homogeneity with grain sizes of ~35–40 nm throughout the disk material. A color-coded contour hardness map with a set of unique color keys that denote values from 180 to 360 provide the homogeneous hardness distribution with an average of 340 across the Al-Mg disk after 100 HPT turns.

Figure 1 shows the cross-sections of the Al-Mg disks (upper) after HPT for 1 turn as an example displaying the typical bonded sample^9^ and for 100 turns with the micrographs taken by a transmission electron microscope (TEM) at the disk center and edge (center) and a color-coded contour hardness map for the material after 100 HPT turns (lower). From the cross-section after 100 turns, it is apparent that there is no evidence of a Mg-rich phase across the disk diameter of the severely deformed material where the Mg-rich phase in the bonded material tends to appear in a darker color at the mid-plane as seen in the 1-turn sample. The current HPT procedure maintained a constant volume of material without any significant change during 1 to 100 turns by HPT, thereby implying the presence of reasonably consistent volume fractions of Mg in the Al-Mg system after HPT from 1 to 100 turns. It should be noted that segregation and a crack are visible at the lower left of the severely processed disk but this occurred during the molding and cutting after the HPT processing.

The microstructural homogeneity is visible by the TEM micrographs where there is a consistent nano-scale microstructure with equiaxed grains with grain sizes of ~40 nm and ~35 nm at the center and edge of the disk after 100 HPT turns, respectively. The corresponding selected-area electron diffraction (SAED) patterns at both the disk center and edge (Extended Data Fig. 1) show spot-distributions on Debye-Scherrer rings indicating the polycrystalline aspect of ultrafine grain sizes within the illuminated volume, indexed solely as 111, 200, 220 and 331 reflections of an *fcc* structure corresponding to Al. There are no diffraction spots indicating the presence of *hcp* Mg and any intermetallic phases, such as Al_3_Mg_2_ and Al_12_Mg_17_, in the diffractograms. In addition, these TEM observations provide little evidence for the existence of deformation twinning in the bulk Al-Mg alloy.

The Vickers microhardness distribution over the vertical cross-section demonstrates a reasonably homogeneous hardness distribution within the material, thus confirming the microstructural homogeneity after HPT for 100 turns on the Al-Mg alloy. In practice, an average hardness of *H*_*v*_ ≈ 340 and a maximum hardness of ~370 were recorded within the disk. Comparing the general saturated hardness ranges of ~65 for a commercial purity aluminum Al-1050 and ~105 for the magnesium ZK60 alloy after HPT, the processing through 100 turns introduced an extreme hardness of the mechanically-bonded Al-Mg alloy. The results provide the first demonstration of a homogeneous microstructure and hardness distributions within a disk material produced by mechanical bonding through HPT. Thus, the microstructural homogeneity contrasts with earlier reports for the Al-Mg^8–10^, Al-Cu^6,7,12^, Mg-Zn^13^ and steel-vanadium^16^ systems.

**Figure 2:**
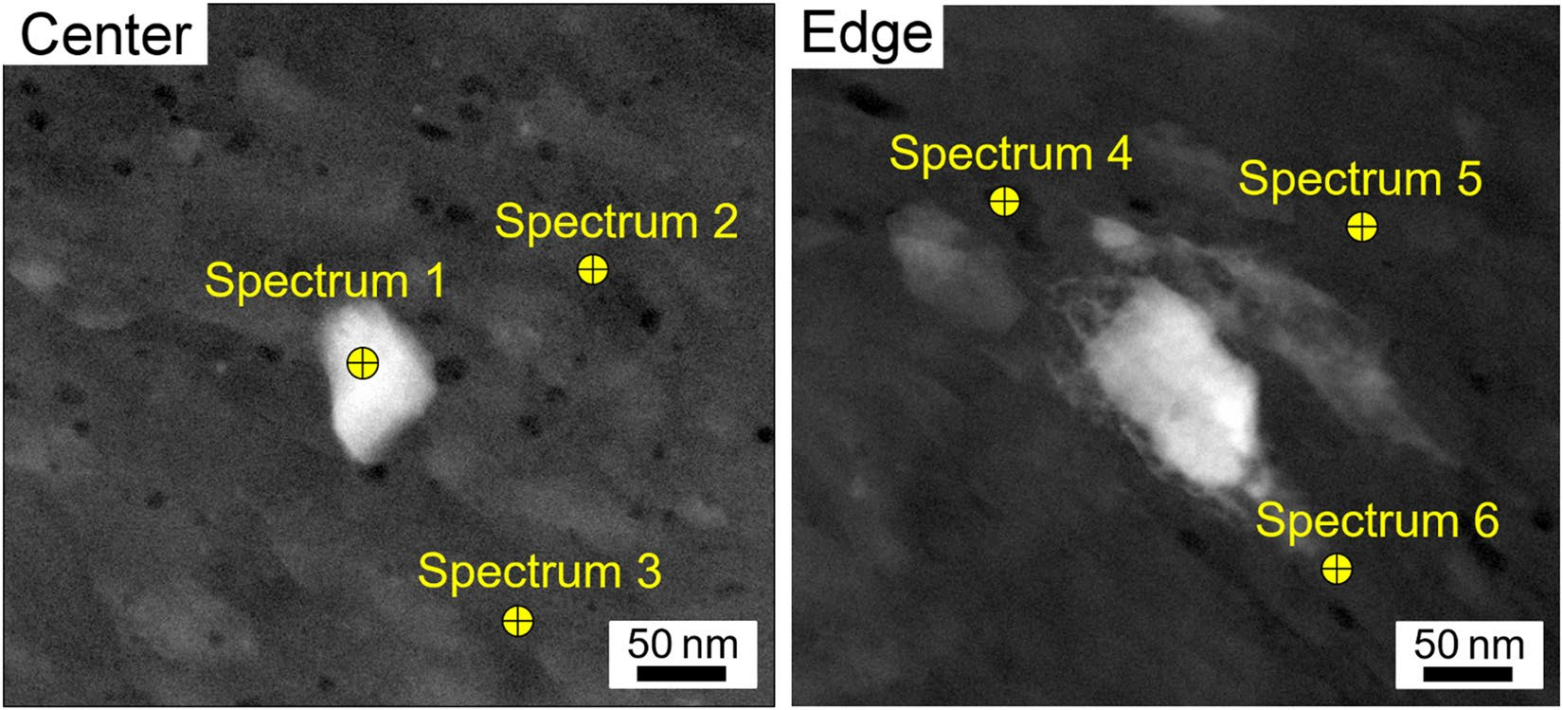
Representative STEM images for the Al-Mg system after HPT. These images are taken from the center and edge of the Al-Mg disk after HPT for 100 turns. The marks describing the examined locations of Spectrums 1–3 and 4–6 from the center and edge regions, respectively.

A compositional analysis was conducted through TEM at both the center and the edge of the bonded Al-Mg material. Figure 2(a,b) shows the representative scanning tunnel microscope electron microscopy (STEM) images with the marks describing the examined locations of Spectrums 1–3 at the disk center and Spectrums 4–6 at the disk edge, respectively, and the associated chemical compositions at the measured locations are listed in Table 1. The corresponding energy dispersive X-ray (EDS) spectra are also available (Extended Data Fig. 2). Widely scattered Mg contents were observed depending on the arbitrarily selected locations. Nevertheless, all locations showed exceptionally high contents of Mg in the Al matrix at room temperature and the detected maximum Mg content was 38.44 at.% which is extremely high by comparison with the solubility limit of Mg of ~1 at.% in Al at room temperature.

**Table 1:** Chemical compositions measured at center and edge of the Al-Mg disk after HPT for 100 turns.

	Chemical composition (atomic%) at the specific measurement location (Spectrum)					
	1	2	3	4	5	6
Al	94.43	65.83	74.24	61.56	84.82	87.59
Mg	5.57	34.17	25.76	38.44	15.18	12.41

The compositions of Al and Mg are examined at the six specific locations denoted in Fig. 2. The units are in atomic percent.

**Figure 3:**
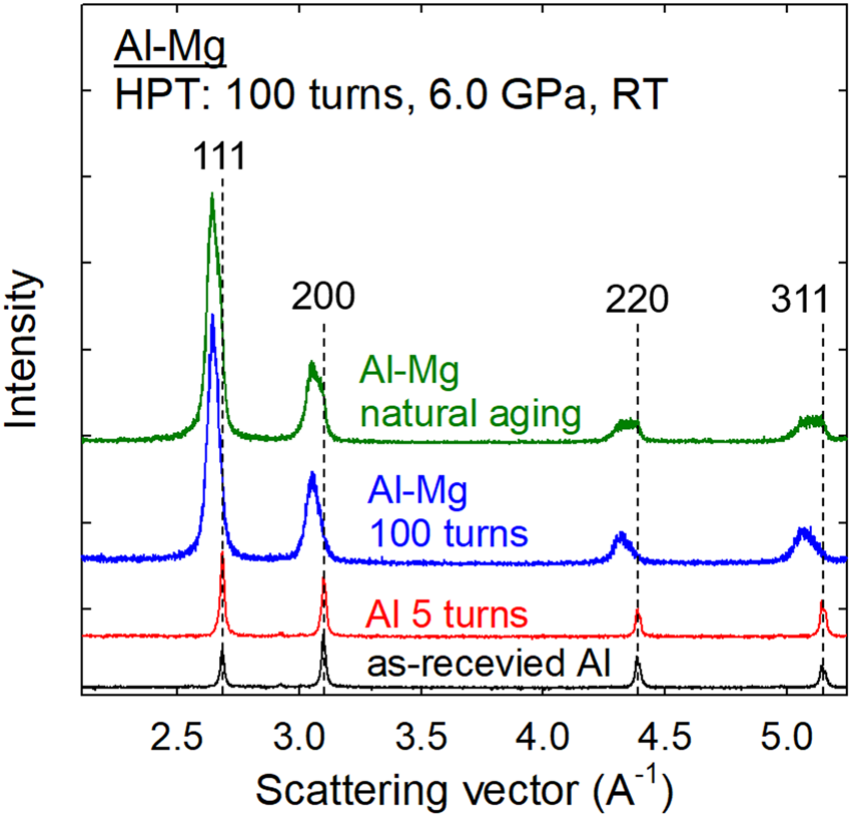
Representative X-ray diffraction profiles. These were taken from a disk surface of the Al-Mg system after HPT for 100 turns and after natural aging. Comparison with the reference XRD profiles for an as-received Al and the Al after HPT for 5 turns suggests that the Al-Mg system after HPT for 100 turns and after natural aging for 60 days has a pure *fcc* structure with significant peak shifting influenced by grain refinement and compositional straining.

The X-ray diffraction (XRD) line profiles for the mechanically bonded Al-Mg alloys are available in earlier reports where these studies show the diffraction peaks of an Al_12_Mg_17_ intermetallic compound^9,10^. Accordingly, the XRD line profiles are shown in Fig. 3 with the scattering vector, *Q*, for, from the bottom, the initial Al sample in the as-received condition, an Al disk processed by HPT for 5 turns, the Al-Mg system immediately after HPT for 100 turns and Al-Mg processed by HPT for 100 turns followed by natural aging at room temperature for 60 days. From the XRD line profiles, it is possible to reach the following three conclusions.

Firstly, the Al-Mg system both after 100 turns by HPT and after natural aging shows consistent diffraction peaks for a pure *fcc* structure, such as 111, 200, 220 and 331, thereby demonstrating a fully Al-based solid solution without any trace of *hcp* Mg or intermetallic phase. Since the material contains a supersaturation of Mg at room temperature, these results indicate that the mechanically-bonded Al-Mg system ultimately forms a nanostructured metastable^17^ alloy in a supersaturated solid solution state of Al after processing by HPT for 100 turns. This compositionally metastable material in a supersaturated solid solution state is maintained stable without any decomposition of a second phase during natural aging for 60 days at room temperature.

Secondly, the nanostructured metastable Al-Mg alloy immediately after HPT for 100 turns and after natural aging show significant shifting of the diffraction peaks to lower scattering angles by comparison with the regular peak locations for pure Al indicated by the vertical broken lines in Fig. 3 where there is no change in the peak position between the as-received Al and the Al after 5 HPT turns. Thus, a large expansion of the Al lattice is anticipated in the metastable materials due to the supersaturation of Mg. Similar peak shifts were reported earlier not in bulk metals but in mechanically alloyed powders produced by ball milling of Al-40 at.% Mg^18,19^. A further analysis by Materials Analysis Using Diffraction (MAUD) computed the mean lattice parameter of the metastable Al-Mg alloy as *a* = 4.1125 Å. Based on Vegard’s law^20,21^, and considering an experimental study on the lattice widening by Mg solutes in Al^22^, the following relationship was obtained for estimating the Mg solubility, *X*_Mg_, in at.% in Al by comparison with the lattice parameter of pure Al, *a*_0_ = 4.049 Å^23^.

1 $${X}_{Mg}=9.045(\frac{a-{a}_{0}}{{a}_{0}})\pm 0.003$$

Thus, Eq. (1) gives an estimated average concentration of Mg in the Al matrix throughout the entire disk of 14.2 at.%. This value is remarkably high when using a route involving a bulk-state reaction without applying an elevated processing temperature.

**Figure 4:**
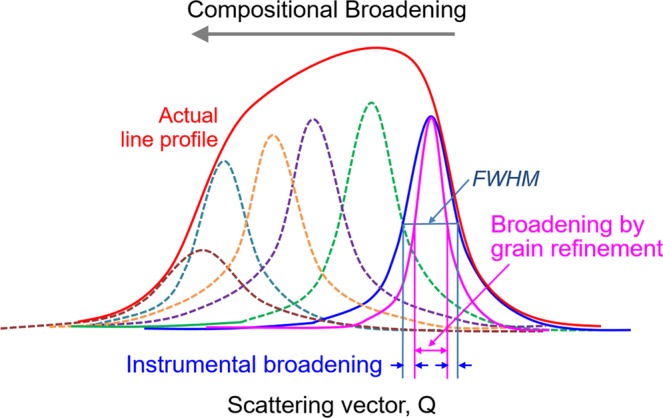
Schematic illustration of the peak broadening in an XRD line profile. This is due to the different components of compositional and instrumental broadening and broadening by grain refinement.

Thirdly, the XRD peaks are significantly broadened for the HPT-induced metastable Al-Mg alloy and in addition the aged material shows these peaks in a plateau configuration which becomes more apparent at the higher scattering angles. This broadening is attributed not only to grain refinement but also to an instrumental broadening due to inherent imperfections in the camera and recording devices and compositional straining where solute atoms expand or compress the matrix lattice^24^. Regarding the large and non-Lorentzian shaped total peak profile due mainly to the distribution of chemical strain where a schematic illustration is shown in Fig. 4, the Williamson-Hall method breaks down to extract a small grain size broadening. Instead, a plateau-shape with a sharp fall-off profile was observed at Δ*a*/*a*_0_ = 0 or *X*_Mg_ = 0 in the metastable solution after aging. However, a tail exists beyond the Al edge to larger scattering vectors or negative strain values which can be attributed to size-broadening. Fitting only the high-*Q* tail to a Lorentzian, a consistent grain size may be estimated from all four reflections. Thus, from a combination of many different peaks, it is reasonable to take the outline of the apparent line profile at the highest scattering vector indicating the smallest lattice parameter which is close to pure Al with a constrained state. Accordingly, the folding of the line fits well with a Lorentzian function^25^ and permits a convolution of a resultant line profile^26^. By performing this analysis, the line broadening of this specific high-*Q* tail of the line profile incorporates the instrumental and grain refinement components without any compositional gradient broadening.

The analyzed data are summarized in Table 2 for the apparent full width at half maximum (FWHM) of the overall line profile with plateau, the FWHM of the unfolded line profile, instrumental broadening estimated on a LaB_6_ from NIST using a similar set-up^27^, the FWHM for the actual grain refinement broadening and the computed crystallite sizes for the four plane reflections of 111, 200, 220 and 311. It is apparent that the instrumental broadening in the present study may have only a limited influence on the overall FWHM at each coordinate. Moreover, the overall FWHM is significantly larger than the actual broadening by the reduced grain sizes and thus the metastable Al alloy after natural aging exhibits significant compositional broadening. The crystallite sizes of ~25–34 nm were estimated after the deconvolution of the line broadening where the line broadening by grain refinement is anticipated as independent of the magnitude of the scattering vector. The result is reasonably consistent with the TEM observations taken at both the center and edge of the Al immediately after HPT for 100 turns as shown in Fig. 1 while the actual grain size of the aged material is expected to be slightly larger than this crystallite size. Nevertheless, it is reasonable to conclude that the nanostructured metastable Al is capable of retaining a consistent grain size for at least two months of natural aging at room temperature.

**Table 2:** The apparent FWHM of the overall line profile with plateau, FWHM of the unfolded line profile, instrumental broadening based on LaB_6_ from NIST^27^, FWHM for actual grain refinement broadening and the computed crystallite sizes for four plane coordinates of 111, 200, 220 and 311.

Plane coordinate	FWHM (overall) (${\AA }^{-1}$)	FWHM (the unfolded line profile) $({\AA }^{-1})$	Instrument broadening (LaB_6_ from NIST^27^) $({\AA }^{-1})$	Actual grain refinement broadening (${\AA }^{-1}$)	Crystallite size (nm)
111	0.0699	0.027	0.0064	0.020	30.0
200	0.0821	0.029	0.0062	0.023	27.2
220	0.1085	0.024	0.0059	0.018	33.5
311	0.1381	0.030	0.0056	0.025	25.0

**Figure 5:**
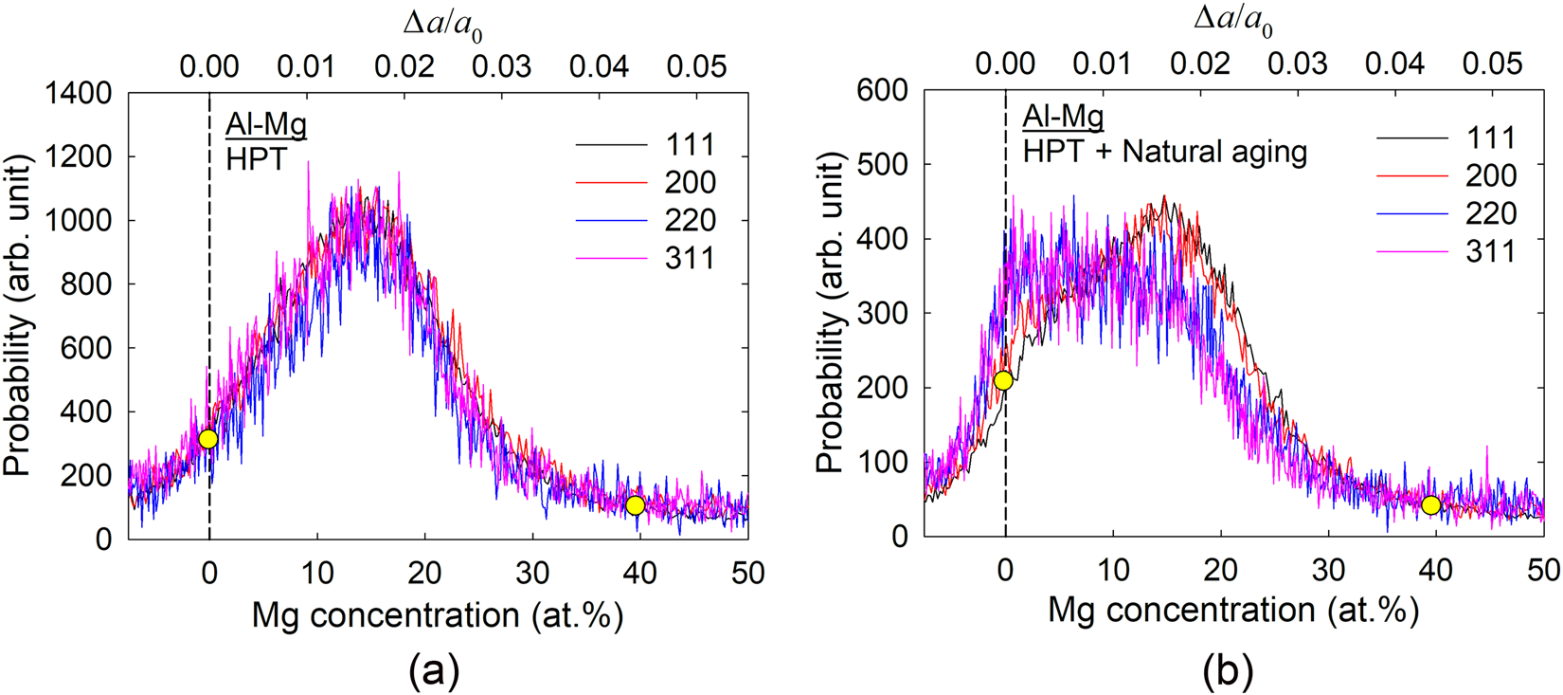
Estimation of the Mg concentrations and compositional strain. The estimated Mg concentration and the compositional strain are shown for the four separate XRD peak reflections for the metastable Al after HPT for 100 turns and after natural aging in (**a,b**), respectively. The vertical dashed lines are calculated with the lattice parameter of *α*_0_ = 4.049 Å for pure Al in each plot.

Further evaluating the strengthened plateau behavior in the XRD line profile for the aged metastable material, and by applying Eq. (1) with arbitrarily selected counting probability, the four separate XRD peaks are visualized in Fig. 5(a,b) for the metastable Al alloy immediately after processing and after natural aging, respectively, where the top abscissa measures the lattice deviation from pure Al in the total compositional strain by Δ*a*/*a*_0_ for any of the scattering vectors of the four observed reflections. This strengthened broadening confirms a heterogeneous Mg concentration in many different nano-scale grains of the metastable alloy. The yellow dots mark the minimum and maximum Mg concentrations estimated from the plots. Thus, the metastable Al alloy immediately after HPT for 100 turns contains Mg solute for a maximum of ~40 at.% where this result is in excellent agreement with the direct observation of 38.44 at.% by chemical analysis and thus the bulk nanostructured metastable Al-Mg alloy contains a supersaturation of Mg solutes. Furthermore, the range of the Mg concentration is maintained reasonably constant during natural aging in the material while the distribution shifts to stronger plateau-shaped curves.

The present results demonstrate the unexpected feasibility of forming a metastable state in a bulk engineering alloy during a nanostructuring process at room temperature to very high strains whereas the current technology of powder metallurgy and powder compaction through HPT introduces only a small volume of a metastable phase within bulk materials^28–30^. In practice, a metastable phase involving amorphous structure is achievable after extreme straining by ultra-severe plastic deformation though HPT on several powder mixture and ingot Mg immiscible alloys^31^, while no data are available for the scale and volume of the metastable phase. Thus, the present study on synthesis of a bulk nanostructured metastable alloy offers significant opportunities for future materials research.

## Methods

### Sample preparation and processing

Two base metals were used in this study: a commercial purity (99.5%) Al-1050 aluminum alloy and a ZK60 magnesium alloy containing 6.0 wt.% Zn and 0.72 wt.% Zr. These materials were received after extrusion to rods with diameters of 10 mm. The rods were sliced into disks with thicknesses of ~1.2 mm and polished on both sides to produce thicknesses of ~0.83 mm. The disks were processed by HPT under quasi-constrained conditions in a monotonic torsional manner at room temperature. Two sets of Al and Mg disks were processed together by HPT with the disks piled on the depression in the lower anvil in the order of Al/Mg/Al (thus, the volume ratio of Al:Mg is 2:1) without any special sample surface treatment^9^ (Illustrations are available in Extended Data Fig. 3). Specifically, when a disk sample is processed in conventional HPT, the shear strain, γ, is computed by a relationship of the form^32,33^

2 $$\gamma =\frac{2\pi Nr}{h}$$

where *N* is the number of revolutions and *r* and *h* are the radius and height (or thickness) of the disk, respectively. The HPT processing was conducted under a compressive pressure of 6.0 GPa for 100 turns at a rotational speed of 1 rpm and thus the edge of the sample receives a maximum shear strain of ~4,000. In addition, one of the disks was taken after processing and naturally aged at room temperature for 60 days to evaluate the microstructural stability. It is reasonable to note that the median plane of the sample stack shows some material loss due to outflow between the anvils and this tends to be excessive for Mg thereby reducing the initial overall composition of 26 at.% Mg from a volume ratio 2:1 to the observed 14.2 at.% Mg.

### Hardness measurements

One of the HPT-processed disks was cut along the diameter and the vertical cross-sectional surfaces of two semi-circular disks were polished mechanically to mirror-like conditions. One of these surfaces was etched with Keller’s etchant for microstructural observations using an optical microscope equipped with a Vickers hardness tester, Mitutoyo HM-200. The other polished surface was unetched and it was used to measure the Vickers microhardness values at intervals of 0.15 mm in a grid pattern with a load of 50 gf (0.49 N) and a dwell time of 10 s.

### Microstructural observations

Detailed microstructural observations were conducted using TEM, JEOL JEOM-2100F, at the center and edge of the disk processed by HPT for 100 turns. The TEM specimens were prepared using a focused ion beam, FEI Quanta 3D FEG, where the samples were taken at the disk center at *r* ≈ 0 mm and at the disk edge at *r* ≈ 4.0 mm, respectively, where *r* is the radius of the disk. High-resolution compositional analysis was conducted using energy dispersive spectroscopy in a scanning TEM mode.

### Texture and compositional analyses

The changes in the crystal structure, chemical compositions, lattice parameter and crystal domain size were examined with XRD analysis using a facility, Rigaku Ultima III, where the measurements integrate the data on the overall horizontal surface of the processed disk. The diffraction patterns were obtained using Cu-K_α_ radiation in a Bragg-Brentano configuration with a scanning speed of 1°/min and a step size of 0.01°. The analytical software package MAUD^34^, which is based on the Rietveld method, was used to identify and quantify the amounts of compounds from these XRD profiles.

## Supplementary information

Extended Data Fig. 1, Extended Data Fig. 2, Extended Data Fig. 3

## Data Availability

The data supporting the findings of this study are available from the corresponding author on request.
