# Supplementary material for: Synthesis of a bulk nanostructured metastable Al alloy with extreme supersaturation of Mg
Source: Sci Rep. 2019 Nov 20;9:17186. doi: 10.1038/s41598-019-53614-3 (PMC6868221; doi:10.1038/s41598-019-53614-3)
Supplement: Supplementary file 1 — Extended Data Fig. 1, Extended Data Fig. 2, Extended Data Fig. 3 [file 41598_2019_53614_MOESM1_ESM.docx]

Supplementary Information

**Synthesis of a bulk nanostructured metastable Al alloy with**

**extreme supersaturation of Mg**

Jae-Kyung Han^1^, Klaus-Dieter Liss^2,3^, Terence G. Langdon^4^, Megumi Kawasaki^1^*

^1^ School of Mechanical, Industrial and Manufacturing Engineering, Oregon State University, Corvallis, OR 97331, U.S.A

^2^ Materials and Engineering Science Program, Guangdong Technion - Israel Institute of Technology, Shantou, Guangdong 515063, China

^3^ Technion – Israel Institute of Technology, Haifa 32000, Israel

^4^ Materials Research Group, Department of Mechanical Engineering,

University of Southampton, Southampton SO17 1BJ, U.K.

**Corresponding author*: Megumi Kawasaki; tel: 541-737-4571; fax: 541-737-2600;

e-mail: [megumi.kawasaki@oregonstate.edu](mailto:megumi.kawasaki@oregonstate.edu)


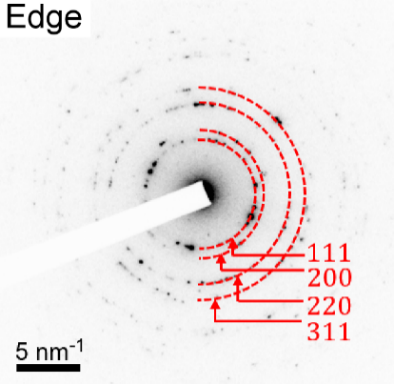

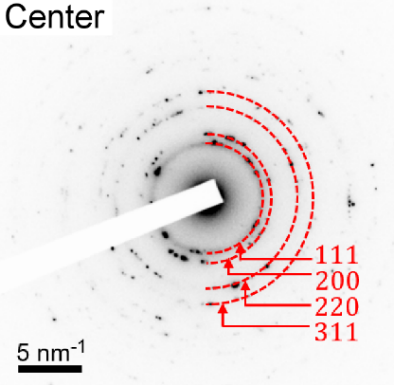


Extended Data Fig. 1 **The SAED patterns for the Al-Mg system after HPT**. These patterns are taken at the disk center and edge regions consistent with the area captured in the TEM images in Fig. 1. These prove that the Al-Mg system shows solely 111, 200, 220 and 331 reflections of an *fcc* structure that correspond to Al.

Extended Data Fig. 2 **Corresponding EDS spectra for the examined locations, Spectrums 1-6, in the Al-Mg system after HPT.** The marks describing the examined locations of Spectrums 1-3 and 4-6 from the center and edge regions, respectively


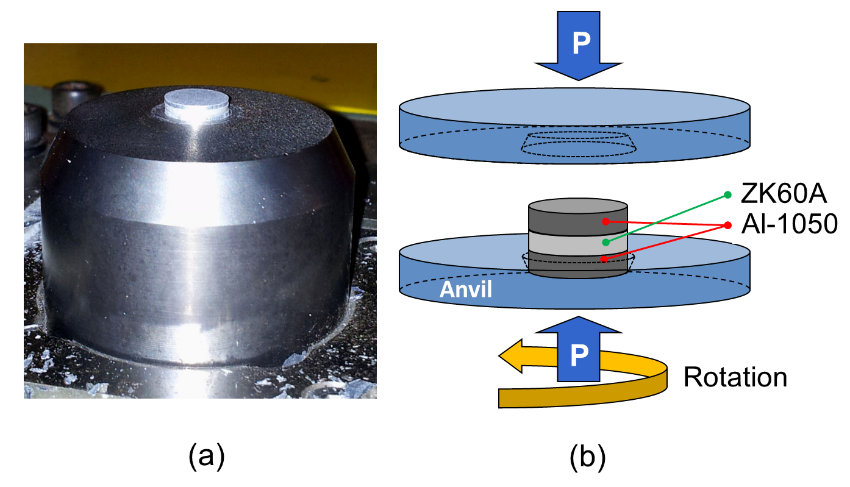


Extended Data Fig. 3 **Illustrations of the unique HPT sample set-up.** (a) Photograph showing a pile of three disks on the lower HPT anvil before processing and (b) schematic illustration of the detailed sample set-up and HPT facility (Ahn *et al.* Mater. Sci. Eng. A 635, 109-117 (2015).
